# Exceptional Long-Term Disease Stability Under Nivolumab in Recurrent High-Grade Glioma: A Case Report

**DOI:** 10.7759/cureus.110942

**Published:** 2026-06-16

**Authors:** Joana N Santos, Francisca S Batista, Frederico Sanches, Ana Miranda, Mariana Santiago, Beatriz Castanheira, J. Guilherme Gonçalves-Nobre, Patricia Lima, Filipe Soares Nogueira, Joao Brito

**Affiliations:** 1 Medical Oncology, Hospital Garcia de Orta, Almada, PRT; 2 Neuroradiology, Hospital Garcia de Orta, Almada, PRT; 3 Oncology, Hospital Garcia de Orta, Almada, PRT; 4 Hemato-oncology, Hospital Garcia de Orta, Almada, PRT; 5 Pathology, Hospital Garcia de Orta, Almada, PRT

**Keywords:** disease stability, high-grade glioma, idh mutation, immunotherapy, long-term survival, mgmt methylation, nivolumab

## Abstract

Immune checkpoint inhibitors have not demonstrated a survival benefit in unselected high-grade glioma populations; however, a subset of patients may achieve durable disease control. We report the case of a 30-year-old male patient diagnosed with a right temporoparietal high-grade glioma, initially classified as anaplastic astrocytoma and later evolving to astrocytoma, isocitrate dehydrogenase (IDH)-mutant, grade 4 according to the 2021 World Health Organization classification. The patient underwent multiple surgical resections followed by radiotherapy with concomitant temozolomide and subsequent adjuvant temozolomide (Stupp protocol). Upon disease recurrence, he received second-line treatment with lomustine plus bevacizumab, with documented progression across successive lines of therapy. In the absence of standard therapeutic options, off-label nivolumab was initiated in November 2018 following further progression. The tumor harbored an IDH1 R132H mutation and O6-methylguanine-DNA methyltransferase (MGMT) promoter methylation. Corticosteroids were discontinued prior to immunotherapy initiation. Serial magnetic resonance imaging demonstrated sustained radiological stability, without evidence of nodular enhancement or increased perfusion suggestive of recurrence. The most recent imaging assessment (March 2026) confirmed ongoing disease stability of more than seven years after treatment initiation. Treatment was well tolerated, with only mild immune-related arthralgias reported. The patient remains functionally independent, with stable mild residual hemiparesis. This case highlights the potential for prolonged disease stability under anti-Programmed cell death protein 1 (PD-1) therapy in selected patients with high-grade glioma. Although immunotherapy has not shown benefit in unselected populations, molecular features such as IDH mutation and MGMT promoter methylation, as well as the absence of corticosteroid use, may contribute to enhanced treatment responsiveness and warrant further investigation.

## Introduction

High-grade gliomas are among the most aggressive primary brain tumors, with limited therapeutic options and poor prognosis, particularly in the recurrent setting. The current standard of care consists of maximal safe surgical resection followed by radiotherapy with concomitant and adjuvant temozolomide, commonly referred to as the Stupp protocol [1]. Despite this multimodal approach, most patients experience disease progression, and outcomes remain unfavorable.

Immune checkpoint inhibitors targeting the programmed cell death protein 1 (PD-1) pathway have demonstrated substantial clinical benefit across multiple solid tumors [2]. However, in high-grade gliomas, their efficacy has been limited. The phase III CheckMate 143 trial did not demonstrate a survival advantage for nivolumab compared to bevacizumab in patients with recurrent disease [3]. This lack of efficacy has been attributed to the unique tumor microenvironment of gliomas, characterized by low immunogenicity and multiple mechanisms of immune suppression.

Nevertheless, survival curves from clinical trials suggest a small subset of patients may experience durable disease control with immune checkpoint inhibitors. This observation supports the hypothesis that specific clinical and molecular features - such as tumor biology and treatment context - may influence responsiveness to immunotherapy, although these factors remain poorly defined.

We report a case of prolonged disease stability exceeding seven years under nivolumab in a patient with recurrent astrocytoma, isocitrate dehydrogenase (IDH)-mutant, grade 4, highlighting the potential for sustained benefit in selected patients.

## Case presentation

A 30-year-old male patient with no relevant past medical history presented in March 2010 with progressive morning headaches, nausea, and vomiting. Neurological examination was unremarkable. Brain magnetic resonance imaging (MRI) revealed a right temporoparietal intra-axial lesion with vasogenic edema and mass effect (Figure 1).

**Figure 1:**
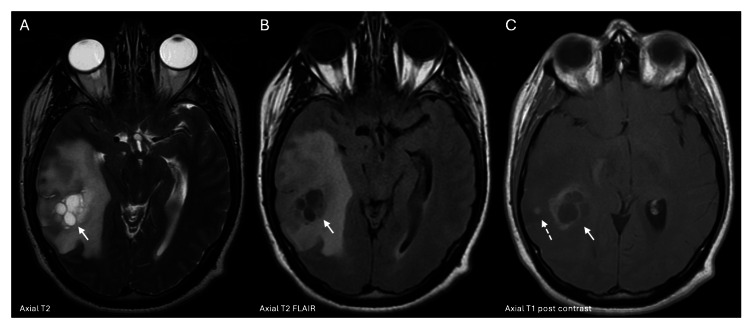
Brain MRI images These were performed in March 2010 and show a right cortico-subcortical temporo-occipital infiltrative lesion with cystic-necrotic components and an associated enhancing satellite lesion. Histopathology confirmed an anaplastic astrocytoma, WHO grade III, according to the 2007 WHO Classification of Central Nervous System Tumors [4]. A: Axial T2-weighted image; B: Axial T2-Fluid-Attenuated Inversion Recovery (FLAIR) image; C: Axial T1 post-contrast image.

The patient underwent right parietal craniotomy with tumor resection and carmustine wafer implantation. Histopathological examination (Figure 2) was consistent with a high-grade glioma, most compatible with anaplastic astrocytoma (WHO grade III, according to the classification in use at the time [4]).

**Figure 2:**
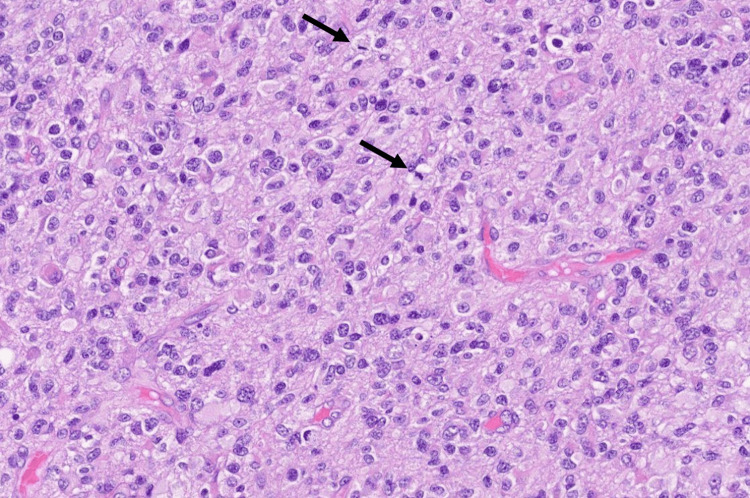
Hematoxylin-eosin-stained section from the initial surgical specimen (2010) Specimen shows high-grade astrocytic features, including hypercellularity, marked nuclear pleomorphism, hyperchromatic and irregular nuclei, and frequent mitotic figures (arrows). These findings supported the diagnosis of anaplastic astrocytoma, WHO grade III.

The patient subsequently completed radiotherapy (60 Gy) with concomitant temozolomide followed by 12 cycles of adjuvant temozolomide. In 2015, imaging demonstrated disease recurrence, and a second surgical resection was performed.

Histopathology (Figure 3) revealed a malignant glioma with features suggestive of progression toward glioblastoma (WHO 2016 classification [5]).

**Figure 3:**
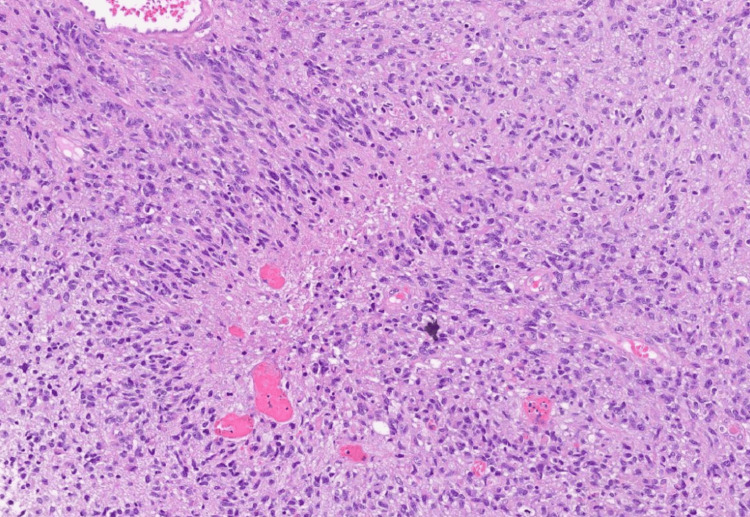
Hematoxylin-eosin stained section Specimen shows palisading necrosis, with tumor cells arranged peripherally around a central necrotic area.

Adjuvant temozolomide was administered.

A second recurrence occurred in 2017, leading to a third surgical resection. Histopathology confirmed glioblastoma, IDH-mutant according to the WHO 2016 classification, corresponding to astrocytoma, IDH-mutant, grade 4 under the current WHO 2021 classification [6]. The patient initiated second-line treatment with lomustine plus bevacizumab, completing six cycles of lomustine, complicated by thrombocytopenia and neutropenia requiring dose modifications, followed by bevacizumab maintenance until August 2018.

In August 2018, imaging demonstrated further disease progression, with recurrent neoplastic involvement of the right inferomedial temporal region anterior to the surgical site (Figure 4).

**Figure 4:**
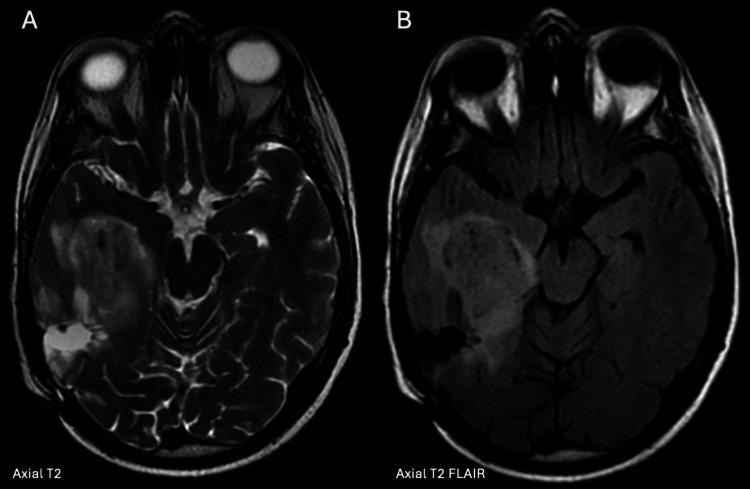
Brain MRI from August 2018 Image shows recurrent neoplastic involvement of the right inferomedial temporal lobe, anterior to the surgical site, characterized by heterogeneous signal intensity on T2-weighted and T2-Fluid-Attenuated Inversion Recovery (FLAIR) images and mass effect.

A fourth surgical procedure with partial resection was performed in September 2018. Histopathology confirmed a high-grade glioma with features consistent with glioblastoma, IDH-mutant according to contemporaneous classification, corresponding to astrocytoma, IDH-mutant, grade 4 under the WHO 2021 classification. Histological features included marked cellularity, nuclear pleomorphism, extensive necrosis, and microvascular proliferation, with a Ki-67 proliferation index of approximately 80%. Immunohistochemistry confirmed IDH1 R132H mutation and p53 overexpression. O6-methylguanine-DNA methyltransferase (MGMT) promoter methylation was documented. PD-L1 expression and mismatch repair status were not available.

The patient had experienced three prior documented radiological progressions before initiation of immunotherapy. Given the absence of standard therapeutic options, the case was discussed at the multidisciplinary tumor board, and off-label nivolumab (3 mg/kg every two weeks) was initiated in November 2018 after written informed consent. Corticosteroids were tapered and discontinued prior to treatment initiation. At baseline, the patient presented with mild left hemiparesis (grade 4+) attributable to surgical sequelae and was undergoing physiotherapy.

After nivolumab initiation, serial MRI studies demonstrated sustained radiological stability. MRI performed in January 2020 showed post-surgical changes without nodular enhancement suggestive of recurrence, and follow-up MRI in February 2026 showed persistent stability without pathological enhancement or increased perfusion (Figure 5).

**Figure 5:**
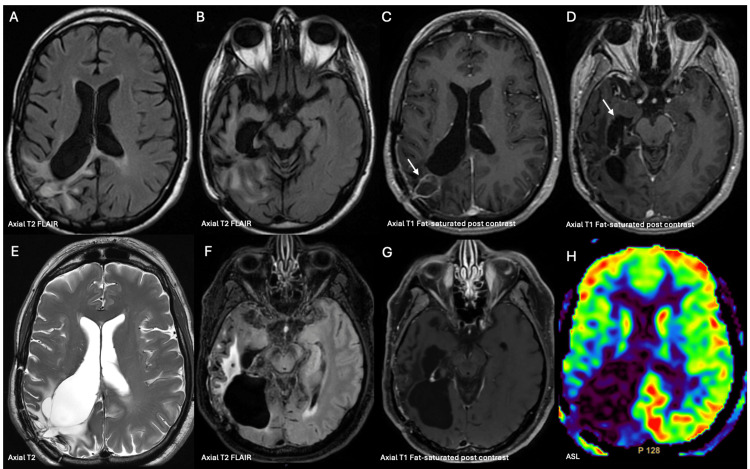
Imaging studies Upper row: Brain MRI from January 2020, one year after the second surgery and following initiation of Nivolumab, demonstrates right temporo-occipital and parietal subcortical hyperintensity on T2-Fluid-Attenuated Inversion Recovery (FLAIR) (A, B), with linear enhancement along the margins of the surgical cavity (C, D – arrows) and mild passive ectasia of the lateral ventricle. No nodular enhancement is identified, arguing against tumor recurrence. Bottom row: Brain MRI from February 2026, during ongoing treatment with Nivolumab, shows progression to gliosis in the affected right hemisphere, with associated increase in lateral ventricular dilatation (E, F). No pathological post-contrast enhancement is observed (G). Perfusion imaging, including arterial spin labeling (ASL, H) and dynamic susceptibility contrast (DSC, not shown), demonstrates no areas of increased cerebral blood flow.

Nivolumab has been maintained continuously for over seven years without dose modifications. Treatment was well tolerated, with only mild immune-related arthralgias reported. At the most recent clinical evaluation, the patient remained functionally independent, with stable mild residual hemiparesis.

A summary of the patient’s clinical course, treatment sequence, and radiological follow-up is provided in Table 1.

**Table 1:** Timeline of clinical course, treatment, and radiological follow-up BCNU: carmustine; GBM: glioblastoma; IDH: isocitrate dehydrogenase; MGMT: O6-methylguanine-DNA methyltransferase; MRI: magnetic resonance imaging; q2w: every two weeks; rCBV: relative cerebral blood volume; RT: radiotherapy; TMZ: temozolomide.

Period	Event	Management / Imaging	Pathology / Notes
March 2010	Initial diagnosis and 1st surgery	Right parietal craniotomy + BCNU wafers	Anaplastic astrocytoma, WHO grade III
July 2010–2011	First-line treatment	RT 60 Gy + concomitant TMZ; 12 adjuvant TMZ cycles	—
April 2015	1st recurrence	2nd surgery + adjuvant TMZ	Malignant glioma; features of GBM transformation
May 2017	2nd recurrence	3rd surgery; lomustine + bevacizumab ×6; bevacizumab maintenance	GBM, IDH-mutant (WHO 2016); astrocytoma, IDH-mutant, grade 4 (WHO 2021)
August 2018	3rd progression	Brain MRI showing further progression	Right inferomedial temporal recurrence
September 2018	4th surgery	Partial resection	GBM, IDH-mutant by historical classification; astrocytoma, IDH-mutant, grade 4 (WHO 2021)
November 2018	Nivolumab initiation	Off-label nivolumab 3 mg/kg q2w; corticosteroids discontinued	Third-line systemic treatment
January 2019	First MRI after nivolumab	Reduced mass effect; no new enhancing lesions	2 months after nivolumab initiation
2019–2025	Serial MRI follow-up	Stable surgical cavities; no nodular enhancement; no rCBV increase	No radiological progression
March 2026	Latest follow-up	Stable disease; mild residual left hemiparesis	>7 years on nivolumab

## Discussion

This case illustrates prolonged disease stability exceeding seven years under nivolumab in a patient with recurrent IDH-mutant, grade 4 astrocytoma. Despite the established role of the Stupp protocol in the upfront management of high-grade gliomas [1], prognosis remains poor in the recurrent setting, with limited therapeutic options.

Immune checkpoint inhibitors targeting the PD-1/Programmed Death-Ligand 1 (PD-L1) axis have demonstrated significant clinical benefit across multiple solid tumors [2], but have not shown a survival advantage in unselected populations with high-grade gliomas. Nevertheless, durable responses have been observed in a small subset of patients [3,7].

The limited efficacy of immunotherapy in gliomas has been attributed to the highly immunosuppressive tumor microenvironment, characterized by low tumor mutational burden, reduced antigen presentation, and increased regulatory T-cell activity [8,9].

Several factors may have contributed to the favorable outcome observed in this case. MGMT promoter methylation is associated with increased sensitivity to alkylating agents and may contribute to higher tumor mutational burden following temozolomide exposure, potentially enhancing tumor immunogenicity [10]. Exploratory analyses from CheckMate 143 suggested a trend toward improved outcomes in MGMT-methylated patients treated with nivolumab, although this did not reach statistical significance [3].

IDH-mutant gliomas represent a biologically distinct subgroup with specific molecular, epigenetic, and clinical characteristics compared with IDH-wildtype tumors. Under the 2021 WHO Classification of Central Nervous System Tumors, IDH-mutant high-grade gliomas are no longer classified as glioblastoma, a term now reserved for IDH-wildtype tumors, and are instead designated as astrocytoma, IDH-mutant, grade 4 [6]. This distinction is particularly relevant when interpreting historical clinical trials such as CheckMate 143, in which patients with IDH-mutant tumors were included under the broader category of glioblastoma.

The absence of corticosteroid use at the time of immunotherapy initiation is also clinically relevant. Corticosteroids impair T-cell function and have been associated with inferior outcomes in patients receiving immune checkpoint inhibitors [7]. Their discontinuation prior to nivolumab initiation in this patient may have preserved the functional capacity of tumor-infiltrating lymphocytes.

Importantly, this patient had experienced multiple documented disease progressions prior to nivolumab initiation, arguing against an intrinsically indolent disease course and supporting a treatment-related effect.

Exceptional responses to anti-PD-1 therapy in high-grade glioma have been described in selected patients, particularly those with underlying mechanisms that increase tumor immunogenicity, such as mismatch repair deficiency or germline predisposition syndromes [11,12]. While no such condition was identified in this case, temozolomide-induced hypermutation represents a plausible contributing mechanism [13].

Limitations of this report include the absence of comprehensive molecular profiling, particularly mismatch repair status, tumor mutational burden, and PD-L1 expression, which may have provided further insight into the mechanisms underlying this response.

## Conclusions

This case reports prolonged disease stability exceeding seven years under nivolumab in a patient with recurrent, IDH-mutant, grade 4 astrocytoma. Although immune checkpoint inhibitors have not demonstrated benefit in unselected populations with high-grade glioma, this case suggests that selected patients may achieve sustained disease control.

The presence of IDH mutation, MGMT promoter methylation, prior exposure to alkylating chemotherapy, and absence of corticosteroid use at immunotherapy initiation may have contributed to the favorable clinical course. Further studies are needed to identify predictive biomarkers and define which patients with high-grade glioma may benefit from immune checkpoint inhibition.
